# King George V

**Published:** 1910-06-04

**Authors:** 


### The Hospital

A Journal of

The Medical Sciences and Hospital Administration.

New Series. No. 172, Vol. VII. [No. 1244, Vol. XLVIII.] SATURDAY, JUNE 4, 1910

### KING GEORGE V.

The Special Hospital Sunday Number, which we shall issue next week, contains the story of King Edward's work for the hospitals, and of the establishment of the King's Hospital Fund. King George, whose birthday was celebrated on Friday, showed the greatest possible interest in his father's work for the hospitals. The interest which he then displayed has never flagged, and when King George became Grand President and Queen Mary Lady Grand President of the League of Mercy in 1901, both devoted themselves to the work with a zeal and energy which inspired all connected with the League, and have had much to do with its phenomenal success. Year by year they have assembled at Marlborough House, at the special garden party which they have given with great liberality, the officers, members, and associates of the League of Mercy, and have exerted themselves on each occasion for some hours to further the influence and success of the League. They have endeavoured to welcome everybody present, and to impress all with the feeling that workers for the League of Mercy, from the highest to the lowest, are comrades in personal service—a fellowship which should make them better and happier subjects of the King. No words could exaggerate the importance of the service which their Majesties have thus rendered to the cause of the voluntary hospitals. Had they confined their efforts to this work alone they would have won the affections and gratitude of the people, but the League of Mercy has been but one of many directions in which their Majesties have shown that, like good King Edward, the cause of the sick is a matter very near their hearts, which commands all their sympathy.

King George became President of the Prince of Wales's Hospital Fund in 1901, the name of which was changed to King Edward's Hospital Fund at the end of that year. He immediately evinced great energy in the work, and to His Majesty belongs the credit of having raised the greater portion of the magnificent sum now invested in connection with that Fund, which brings in a revenue of upwards of £68,000 per annum. In conjunction with Queen Mary he has systematically rendered service in the cause of the sick, and their annual visits and inspections of hospitals have led to important developments and improvements, while they have placed their Majesties in possession of much practical knowledge, so that an inspection by either of them is no perfunctory act, but an occasion of practical value to each institution visited. King George had further much to do with the King Edward's Hospital Fund for London Act, 1907, which has incorporated its Council, provided for its management and the investment of its funds. Under this Act the vacancy caused in the presidency of the Fund by the accession of King George can be filled by some person being a son, brother, or grandson of the Sovereign, who, in the opinion of the Lord Chancellor, the Prime Minister, and the Governor of the Bank of England, is duly qualified to fill this important office. In default of such an appointment three governors are to be appointed who, during the continuance of the vacancy in the office of President, will possess all the powers belonging to the office of President, and two of whom will constitute a quorum. These three governors are to be appointed by the Sovereign on the recommendation of the Lord Chancellor, the Prime Minister, and the Governor of the Bank of England, or a majority of them, and will hold office for five years or until the appointment of a President, and will be eligible for reappointment. The present position, so far as the King's Hospital Fund is concerned, is one of momentous importance, but King George and his advisers may be trusted to make a wise selection which will ensure the continuous progress of the King's Fund. This Fund has won for itself, by wise administration, a position of strength and general acceptance which is as remarkable as it has been fruitful in advantage to the voluntary hospitals of the Metropolis.

There are few hospital managers who possess as sound a knowledge of hospital administration and of the requirements of a modern hospital as is at present possessed by King George himself. He has devoted time, brains, and energy to the acquirement of full knowledge of the questions involved, and whilst his speeches have been remarkable for their generous acknowledgment of services rendered on the part of others, they have displayed a grasp of hospital affairs, and a wisdom in counsel which have led to the introduction of many economic and other improvements. These innovations will leave their mark upon our hospital system, for they have made it much more thorough than ever before. We are confident that although King George must necessarily relinquish the Presidency of the King's Fund, and of several hospitals with which he has identified himself, personal service in the cause of the sick will always receive his warm advocacy. We may rest assured that on every possible occasion he will show that the many good works with which he was connected as heir-apparent will never cease to interest him and his consort.

The expressions of sincere and heartfelt sympathy for the Royal Family which the death of our lamented Sovereign, King Edward VII., have called forth from all classes of his subjects must, we feel confident, inspire King George and Queen Mary with a great desire to leave nothing undone which can at any time promote the best interests of the people. King George should make an ideal King, for the welfare and progress of the Empire have been, and will continue to be, his first consideration. It is a great matter for the English peoples that family affection and family life should have such a firm hold upon the Royal Family. Great as was the success which attended the self-denying and strenuous efforts of his lamented father, we believe that King George, from his intimate knowledge of the thought and habits of the people in every part of the British Empire, is calculated to win for himself such a whole-hearted and continuous support that his reign should prove to be one of the greatest in history. It cannot fail to bring lustre and authority to the British race in every part of the civilised world.

